# TIMP-2 secreted by monocyte-like cells is a potent suppressor of invadopodia formation in pancreatic cancer cells

**DOI:** 10.1186/s12885-019-6429-z

**Published:** 2019-12-13

**Authors:** Christian Benzing, Hoyin Lam, Chi Man Tsang, Alexander Rimmer, Yoana Arroyo-Berdugo, Yolanda Calle, Claire M. Wells

**Affiliations:** 1 School of Cancer and Pharmaceutical Sciences, New Hunts House, Kings College London, London, SE1 1UL UK; 2 Department of Surgery, Campus Charité-Mitte and Campus Virchow-Klinikum, Charité-Universitätsmedizin Berlin, Berlin, Germany; 3 Department of Anatomical and Cellular Pathology and State Key Laboratory of Translational Oncology, The Chinese University of Hong Kong, Hongkong, Hong Kong SAR; 4 Department of Life Sciences, University of Roehampton, London, UK

**Keywords:** Pancreatic ductal adenocarcinoma, Monocytes, Tumor microenvironment, TIMP2, Invadopodia

## Abstract

**Background:**

Monocytes are a major component of the tumor microenvironment (TME) in pancreatic ductal adenocarcinoma (PDAC). However, the complex interactions between tumor cells and monocytes and their role in tumor invasion have not been fully established.

**Methods:**

To specifically test the impact of interaction on invasive potential two PDAC cell lines PaTu8902 and CFPAC-1 were selected on their ability to form invasive adhesions, otherwise known as invadopodia and invade in a spheroid invasion assay.

**Results:**

Interestingly when the PDAC cells were co-cultured with undifferentiated THP1 monocyte-like cells invadopodia formation was significantly suppressed. Moreover, conditioned media of THP1 cells (CM) was also able to suppress invadopodia formation. Further investigation revealed that both tissue inhibitor of metalloproteinase (TIMP) 1 and 2 were present in the CM. However, suppression of invadopodia formation was found that was specific to TIMP2 activity.

**Conclusions:**

Our findings indicate that TIMP2 levels in the tumour microenvironment may have prognostic value in patients with PDAC. Furthermore, activation of TIMP2 expressing monocytes in the primary tumour could present a potential therapeutic opportunity to suppress cell invasion in PDAC.

## Background

Pancreatic ductal adenocarcinoma (PDAC) is a devastating disease characterised by an aggressive biological tumour behaviour leading to a high mortality rate [1]. The invasive character of PDACs leads to a rapid progression of the disease [2]. In order to invade the surrounding tissue or to create distant metastases, cancer cells need to break through the basement membrane and degrade the extracellular matrix. One identified strategy is to utilise actin-rich membrane protrusions called invadopodia [3] that can degrade extracellular matrix. Initiating mechanisms to suppress such formation is a potential therapeutic target [4]. Unlike podosomes - which are present in normal myeloid cells – invadopodia are specific for invasive cancer cells [5]. The primary purpose of invadopodia is the targeted secretion of matrix metalloproteases (MMPs) to degrade the extracellular matrix (ECM) [6]. Membrane-type 1 metalloprotease (MT1-MMP, also known as MMP14), MMP9 and MMP2 have been identified as the most important MMPs in invadopodia functionality [7, 8].

MMPs can be inhibited by tissue inhibitors of metalloproteinases (TIMPs). Of the four TIMPs [9, 10], TIMP1 and 2 are the best-studied. TIMP1 has a relatively low affinity for membrane-bound MMPs [11] whereas TIMP2 is a strong inhibitor of MT1-MMP [12]. Very little is known about the effects of TIMPs on invadopodia formation, although one study in human breast cancer cells suggested TIMP2 is specifically able to decrease the formation of invadopodia [13].

In recent years, the tumour microenvironment (TME) has become of major interest to researchers and has recently been suggested to play a specific role in influencing the formation of invadopodia [14]. Around 90% of the PDAC mass are stromal cells, only 10% are carcinoma cells [15]. The main components of the TME are cancer-associated fibroblasts (CAF) and tumour-associated macrophages (TAMs). The pancreatic TME is rich in TAMs but undifferentiated monocytes [16] are also present at detectable levels. The interaction between monocytes / macrophages and the tumor cells is complex with both pro- and anti-tumorigenic effects reported [17].

This study aimed to establish whether an interaction between PDAC cells and undifferentiated monocyte-like cells presented pro- or anti-tumorigenic responses.

## Methods

### Cell culture

Patu8902 and Capan2 were obtained from “Deutsche Sammlung von Mikroorganismen und Zellkulturen GmbH” [DSMZ], Germany), Capan1 and MiaPaCa2 were kindly provided by Prof. H. Kocher at Barts Cancer Institute, UK. The human breast cancer cell line MDA-MB-231 was purchased from ATCC. All these cells were maintained in Dulbecco’s modified Eagle’s media (DMEM, Sigma Aldrich UK) supplemented with 10% v/v fetal bovine serum (FBS) and 1 mM penicillin/streptomycin. CFPAC-1 cells (kindly provided by Dr. A. Pessina at Universita degli studi di Milano, Italy) were cultured in Iscove’s modified Eagle’s media (IMEM, Sigma Aldrich UK) supplemented with 10% v/v fetal bovine serum (FBS) and 1 mM penicillin/streptomycin. Colo-357 (kindly provided by Prof. Michalski University Hospital Heidelberg, Germany) and AsPC1 (kindly provided by Dr. Stéphanie Kermorgant, Barts Cancer Institute, UK) were cultured in Roswell Park Memorial Institute-1640 (RPMI-1640, Sigma Aldrich UK) media supplemented with 10% v/v FBS with 1 mM penicillin/streptomycin. THP-1 cells (obtained from the European Collection of Authenticated Cell Cultures catalogue number 88081201) were tagged with enhanced green fluorescent protein (eGFP) using lentiviral vector technique as described before [18]. Cells were maintained in RPMI-1640 media supplemented with 10% v/v FBS with 1 mM penicillin/streptomycin. For inhibitor treatment cells were incubated with 10 mM GM6001 as previously described [19]. The cell lines have not recently been authenticated. All cell lines were regularly screened for mycoplasma contamination.

### THP-1-conditioned media (CM)

THP-1 cells were seeded in serum-free, antibiotic-free RPMI media at a density of 200,0000 cells/ml and incubated at 37 °C and a 5% carbon dioxide (CO_2_) atmosphere. After 24 h, the cell suspension was centrifuged at 1500 rpm at room temperature for 10 min. CM was either directly used for experiments (fresh CM), stored at − 20 °C and rewarmed to 37 °C (frozen CM) or heat-inactivated at 95 °C for 10 mins (boiled CM).

### Antibodies

Anti-TIMP1 antibody (D10E6) produced in rabbit was purchased from Cell Signalling Technology, U.S.A., anti-TIMP2 antibody produced in rabbit antibody (SAB4502972) was purchased from Sigma. Anti-MT1-MMP, Anti-MMP2 and Anti-MMP9 antibody were produced in rabbit and purchased from Cell Signalling Technology, U.S.A.. Anti-GAPDH antibody was purchased from Millipore, U.S.A.

### Invadopodia assay

For the invadopodia assays, the QCM™ Gelatin Invadopodia Assay (Red) (Chemicon® / Millipore) was used. Briefly, coverslips were inverted onto poly-L-lysine in deionized water for 20 min at room temperature (RT). The slides were then washed with phosphate buffered saline (PBS) three times before incubation with glutaraldehyde: PBS for 15 min at RT. After washing three times with PBS, each coverslip was placed on gelatin in PBS in a 1:5 ratio of fluorescently-labelled - unlabelled gelatin and incubated for 10 min at RT and subsequently washed in PBS three times. The. Patu8902 and CFPAC-1 cells were detached using non-enzymatic Cell Dissociation Solution (Sigma Aldrich UK), resuspended in DMEM F-12 growth media (10% FBS, 1 mM penicillin/streptomycin) and seeded onto the prepared coverslips. For co-culture experiments cells were seeded in the presence of control media (serum-free RPMI media and DMEM-F12 media in a 1:1 ratio), in the presence of THP1-CM (frozen CM mixed with DMEM-F12 media with 10% FBS and 1% P/S in a 1:1 ratio) or in the presence of 50,000 THP1 cells. Alternatively, PDAC cells were co-cultured with THP1 cells for 24 h prior to dissociation and seeding on prepared coverslips.

### Spheroid assay

Spheroids were formed in black walled 96-wells clear black round bottom ultra-low attachment spheroid microplates (Corning). First, 1000 cancer cells were seeded in 200 μl DMEM F-12 growth media (10% FBS, 1 mM penicillin/streptomycin), Then, the was centrifuged at 200 g for 8 min at room temperature. Cells were then cultured at 37 °C with 5% CO2 for 72 h. After spheroid assembly was achieved, 170ul medium was removed by multichannel pipetting. A collagen mixture was prepared on ice with a final concentration of 1.3 mg/ml rat tail collagen I (Corning). A collagen matrix of 100 μl was then added to each well with a multichannel pipette. The spheroid plate was then incubated at 37 °C with 5% CO2 for 2 h. A 1:1 mixture of serum-free RPMI and DMEM-F12 with 10% FBS and 1 mM penicillin/streptomycin was then added on top of the collagen matrix to initiate the assay. Brightfield images were obtbained with a 4x objective at 0 h and 48 h, respectively.

### MTT viability assay

For the MTT assay, Patu8902 cells in full growth media (DMEM supplemented with 10% FBS and 1 mM penicillin/streptomycin) were seeded in a triplicates in three 96 well plates. Plate 1 was used as a treatment-naïve control. Growth medium in plate 2 and 3 were removed from the wells and replaced with a) normal growth medium (DMEM supplemented with 10% FBS and 1 mM penicillin/streptomycin), b) DMEM supplemented with 10% FBS and 1 mM penicillin/streptomycin mixed with serum-free DMEM (1:1), c) (DMEM supplemented with 10% FBS and 1 mM penicillin/streptomycin mixed with frozen and rewarmed THP1-CM (1:1), d) DMEM supplemented with 10% FBS and 1 mM penicillin/streptomycin containing rTIMP2 (50 ng/ml). The MTT assay is performed after 24 h (plate 1, control), 72 h (plate 2) and 120 h (plate 3). Cells were washed twice with sterile PBS and incubated with MTT:DMEM solution (500 μg/ml) for 4 h. The MTT:DMEM was removed and DMSO added, the plates were then incubated at 37 °C for 10 min. Absorbance was measured at a wavelength of 562 nm using a Nanodrop.

### Western blot

Serum-free THP-1 conditioned media (CM), serum-free RPMI containing recombinant TIMP1 and TIMP2 (expressed in CHO cells Sigma Aldrich UK) in various concentrations ranging from 5 to 6000 ng/ml, respectively, and serum-free RPMI control media, respectively, were filled in a Spin-X® UF concentrators (Spin-X UF 6 10 K MWCO, Corning) and centrifuged at 4000 rpm for 18 min at RT. Subsequently, gel sample buffer was added to the concentrated CM and the sample was boiled at 90 °C for 3 min. Equal amounts of protein were electrophoresed on 10% sodium dodecyl sulfate (SDS)-polyacrylamide gels then transferred to nitrocellulose membranes as described elsewhere [20]. Nitrocellulose were incubated with primary antibodies using the recommended concentrations and horseradish peroxidase (HRP)-conjugated secondary antibodies (Dako Ltd). Cell lysates were seeded in 6-well plates and cultured for 24 h. Lysates were generated when cells were 70–80% confluent. First, cells were washed with PBS and then lysates were generated with 100 μl NP40 based lysis buffer per well. Lysates were scrapped and centrifuged at 13000 x g for 15 min at 4 °C. The Supernatant was then transferred to an Eppendorf tube and boiled for 3 min at 95 °C in 6x laemmli buffer. Samples were stored at − 20 °C. Equal amounts of protein were then electrophoresed on 7.5% SDS-polyacrylamide gels and immunoblotting was performed as described above.

### Determination of rTIMP1 and rTIMP2 concentrations

Since there are no comparable studies using rTIMP1 and 2, respectively, in an invadopodia model, the approximate concentration had to be estimated. Physiologic TIMP1 and 2 levels, respectively, range between 5 and 1000 ng/ml [21, 22]. In a second step, CM and RPMI control media containing either TIMP1 or TIMP2 in various concentrations ranging from 5 to 6000 ng/ml was concentrated as described above and immunoblotted for TIMP1 and TIMP2, respectively. Autoradiographs were quantified using ImageJ software. The concentration of TIMP2 in the conditioned medium was calculated from the intensity values of the TIMP2 signal in the conditioned medium against the intensity values for recombinant TIMP2 signal. TIMP1 and TIMP2 levels in the CM varied between the experiments and were ranging between 5 and 50 ng/ml. Therefore, these concentrations were used for the later rTIMP1 and rTIMP2 experiments.

### Micro Array

THP1 CM was screened for proteins using the RayBio® C-Series Human Cytokine Antibody Array C5 (RayBiotech Norcross, USA) according to the manufacturer’s protocol.

### Immunofluorescence

Following 24 h-incubation, cells were fixed and stained as previously described. Cells were stained for F-actin (Alexa fluor 488-conjugated phalloidin, Invitrogen),DAPI (Sigma Aldrich UK) and cortactin (Anti-Cortactin (p80/85) Antibody, clone 4F11; Millipore).

### Image analysis

Images were analysed using ImageJ 1.51 h (National Institutes of Health, USA). For gelatine degradation analysis, the total amount of degradation per image was measured in a total of 10 images per tested condition. The amount of degradation was computed automatically using ImageJ. For the spheroid assay, the invasive front was determined as the area of all cells invading from the spheroid. The invasive front was calculated as the area of the invading cells relative to the size of the spheroid after 48 h. Relative spheroid growth was determined by measuring the total area of the spheroids at 0 h and 48 h, respectively.

### Statistical analyses

For data collection and statistical analysis, Microsoft Excel (Microsoft, Redmond, USA) and Prism 5.0 (GraphPad Software, La Jolla, CA, USA) were used. To test for significant differences, the two-tailed Student’s t-tests was used. Data are presented as mean ± standard error of the mean (SEM). A difference was considered significant at *p* < 0.05.

## Results

### Pancreatic cancer cells produce invadopodia

**Fig. 1:**
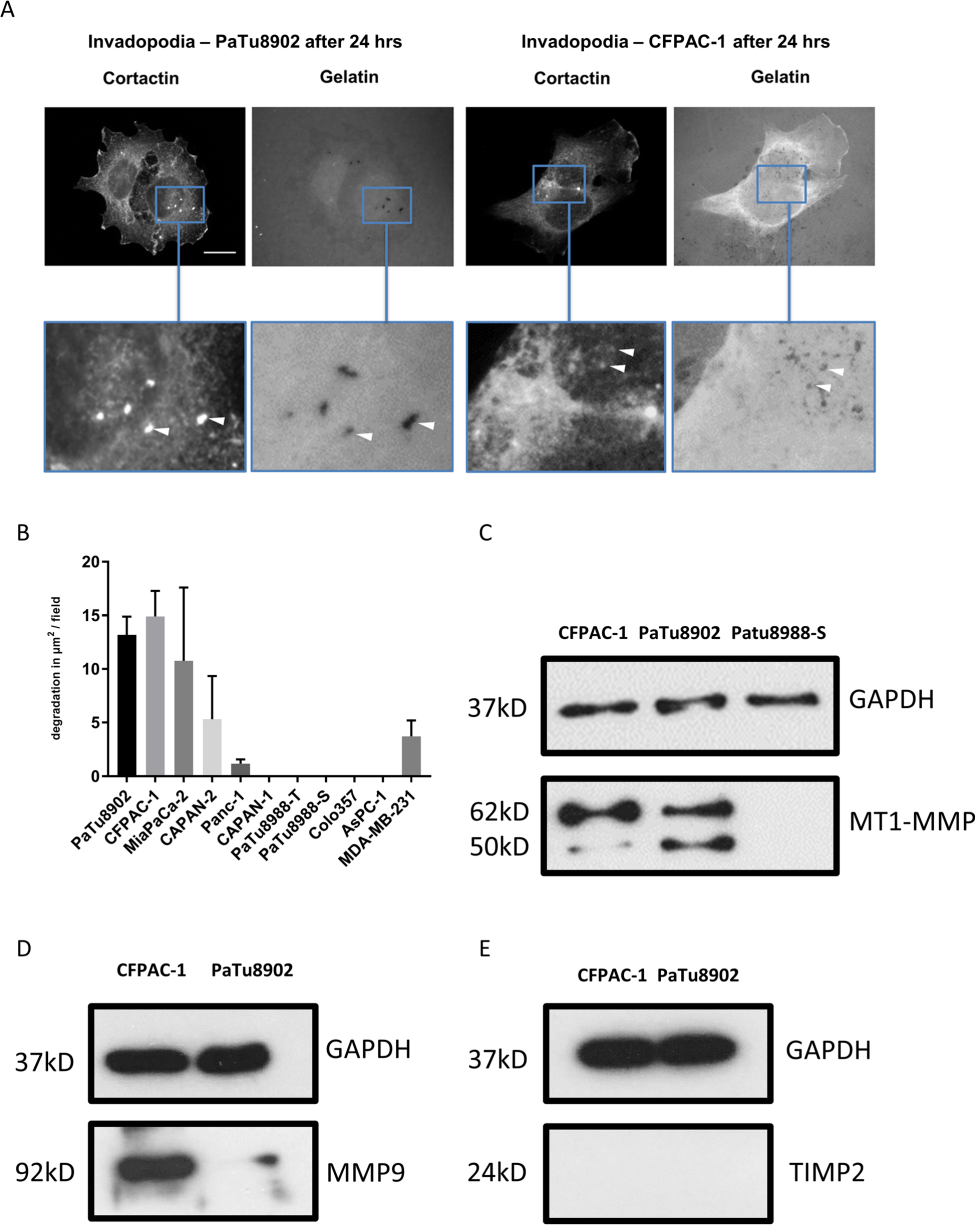
PaTu8902 and CFPAC-1 cells exhibit robust invadopodia activity. **a** Pancreatic ductal adenocarcinoma (PDAC) cell lines were analysed for seeded on fluorescent gelatin for 24 h fixed and stained for cortactin and DAPI. Cortactin puncta corresponding with black dots on the gelatin were considered active invadopodia. **b** Quantification of degradation per field of view for (**a**), MDA-MB-231 cells were also seeded on fluorescent gelatin for 24 h and the degradation per field of view calculated as a positive control. **c** PaTu8902, CFPAC-1 and PaTu8988-S cell lysates were blotted for MT1-MMP. PaTu8902 and CFPAC-1 show high levels of MT1-MMP expression whereas PaTu8988-S shows no MT1-MMP expression. **d** Western blot of PaTu8902 and CFPAC-1 cell lysates showing high levels of MMP9 expression. **e** Western blot of PaTu8902 and CFPAC-1: indicating no expression of TIMP2

As compared to the well-described characteristics of invadopodia formation in breast cancer cells, especially in the cell line MDA-MB 231 [23, 24], less is known about invadopodia in PDAC cells [25]. We screened 10 different PDAC cell lines for invadopodia formation and compared the findings with MDA-MB 231 breast cancer cells (Table 1). Our screen revealed that PaTu8902 and CFPAC-1 constitutively form a large number of invadopodia. To confirm our observations, invadopodia areas of matrix degradation were co-localised with cortactin-positive puncta as a marker of invadopodia (Fig. 1a). We found that PDAC cells require longer incubation times than previously reported for breast cancer cells [23] to generate quantifiable degradation activity and thus quantification of activity was based on the total area of gelatin degradation per field of view. PaTu8902 and CFPAC-1 cells revealed high levels of degradative activity (Fig. 1b). Both invadopodia-forming cell lines PaTu8902 and CFPAC-1 expressed MT1-MMP whereas PaTu8988-S did not (Fig. 1c). CFPAC-1 and PaTu8902 were further tested for MMP9 (positive, Fig. 1d) and MMP2 (no expression, data not shown). Neither CFPAC-1 nor PaTu8902 were expressing TIMP2 (Fig. 1e).

**Table 1:** Tumor characteristics and invadodopodia formation^a^ of tested cell lines

Cell line	Tumor entity	Source of tumour cells	Invadopodia formation
PaTu8902	PDAC	Primary tumor	yes
CFPAC-1	PDAC	Liver metastasis	yes
MiaPaCa-2	PDAC	Primary tumor	inconsistent
CAPAN-2	PDAC	Primary tumor	inconsistent
Panc-1	PDAC	Primary tumor	inconsistent
CAPAN-1	PDAC	Liver metastasis	no
PaTu8988-T	PDAC	Liver metastasis	no
PaTu8988-S	PDAC	Liver metastasis	no
Colo357	PDAC	Lymph node metastasis	no
AsPC1	PDAC	Ascites	no
MDA-MB-231^b^	Breast cancer	Pleural effusion	yes

*PDAC* Pancreatic Ductal Adenocarcinoma

^a^Active invadopodia formation was defined as cortactin puncta corresponding with black dots on the gelatin

^b^MDA-MB-231 cells are well known for producing robust invadopodia formation and were used as a positive control

### Monocyte-like cells co-culture suppresses invadopodia driven matrix degradation

**Fig. 2:**
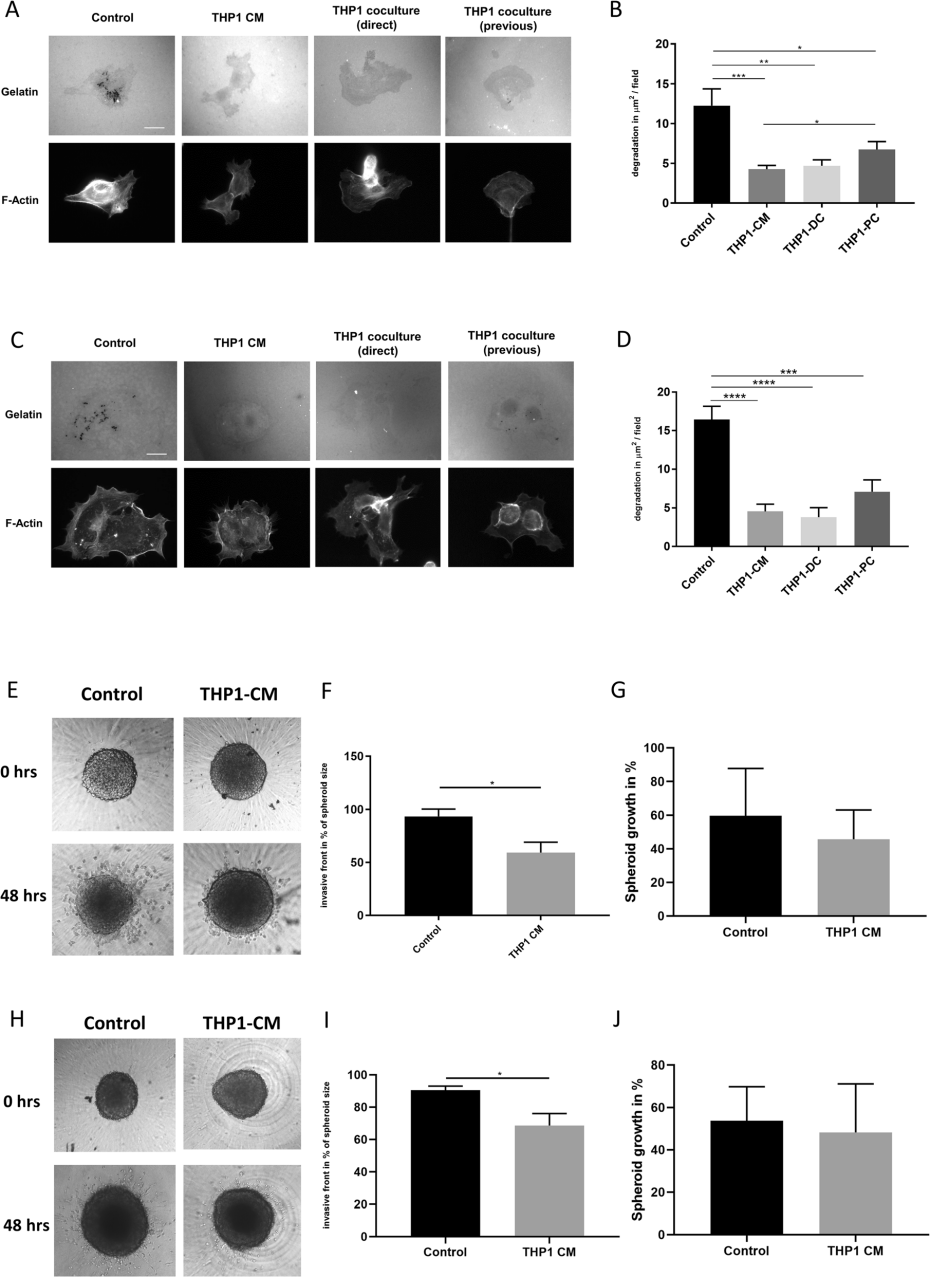
Invadopodia formation can be suppressed by co-culturing PaTu8902 and CFPAC-1 cells with monocyte-like cells. **a** Representative images from a PaTu8902 invadopodia assay where the cells were either incubated with control medium (control), incubated with THP1 conditioned medium (THP1-CM), or were cultured with THP1 cells during the invadopodia assay (THP1-DC), or cultured with THP1 cells prior to the invadopodia assay (THP1-PC). For the THP1-PC condition, growth medium (GM) containing the THP1 cells was evacuated and washed with PBS. **b** Quantification of degradation per field of view for experimental conditions described above (**a**). **c** Representative images from a CFPAC-1 invadopodia assay where the cells were either incubated with control medium (control), incubated with THP1 conditioned medium (THP1-CM), or were cultured with THP1 cells during the invadopodia assay (THP1-DC), or cultured with THP1 cells prior to the invadopodia assay (THP1-PC). **d** Quantification of degradation per field of view for experimental conditions described above (**c**). **e** Representative images from a PaTu8902 spheroid assay where the cells were either incubated with control medium (control) or incubated with THP1 conditioned medium (THP1-CM) at 0 h and 48 h. **f** Quantification of spheroid invasion for experimental conditions described above (**e**). **g** Quantification of relative spheroid growth after 48 h for experimental conditions described above (**e**). **h** Representative images from a Panc1 spheroid assay where the cells were either incubated with control medium (control) or incubated with THP1 conditioned medium (THP1-CM) at 0 h and 48 h. **i** Quantification of spheroid invasion for experimental conditions described above (**h**). **j** Quantification of relative spheroid growth after 48 h for experimental conditions described above (**i**). In all cases **** = *p* < 0.0001, *** = *p* < 0.001, ** = *p* < 0.01, * = *p* < 0.05. Experiments were repeated three times

We performed a number of different co-culture experiments with eGFP-tagged THP1 cells (THP1; a commonly used cell line model for undifferentiated monocyte-like cells) and PaTu8902 cells (Fig. 2a&b) or CFPAC-1 cells (Fig. 2c&d). Either the PDAC cells and THP1 cells were cultured together prior to the invadopodia assay, or PDAC cells and THP1 cells were cultured together during the invadopodia assay, or conditioned medium from THP1 cells was added to the PDAC cells during the invadopodia assay. In all co-culture conditions, gelatin degradation was reduced compared to control in both cell lines (Fig. 2a-d). The suppressive effect of THP-1 CM was confirmed in the spheroid invasion assay. Since CFPAC-1 is not forming adequate spheroids, PANC-1 cells were used instead (Fig. 2e,f,h&i). THP-1 CM did not alter proliferation and spheroid growth (Fig. 2g&j).

### Tissue inhibitor of metalloproteinases 1 and 2 are secreted by THP1 monocyte-like cells

**Fig. 3:**
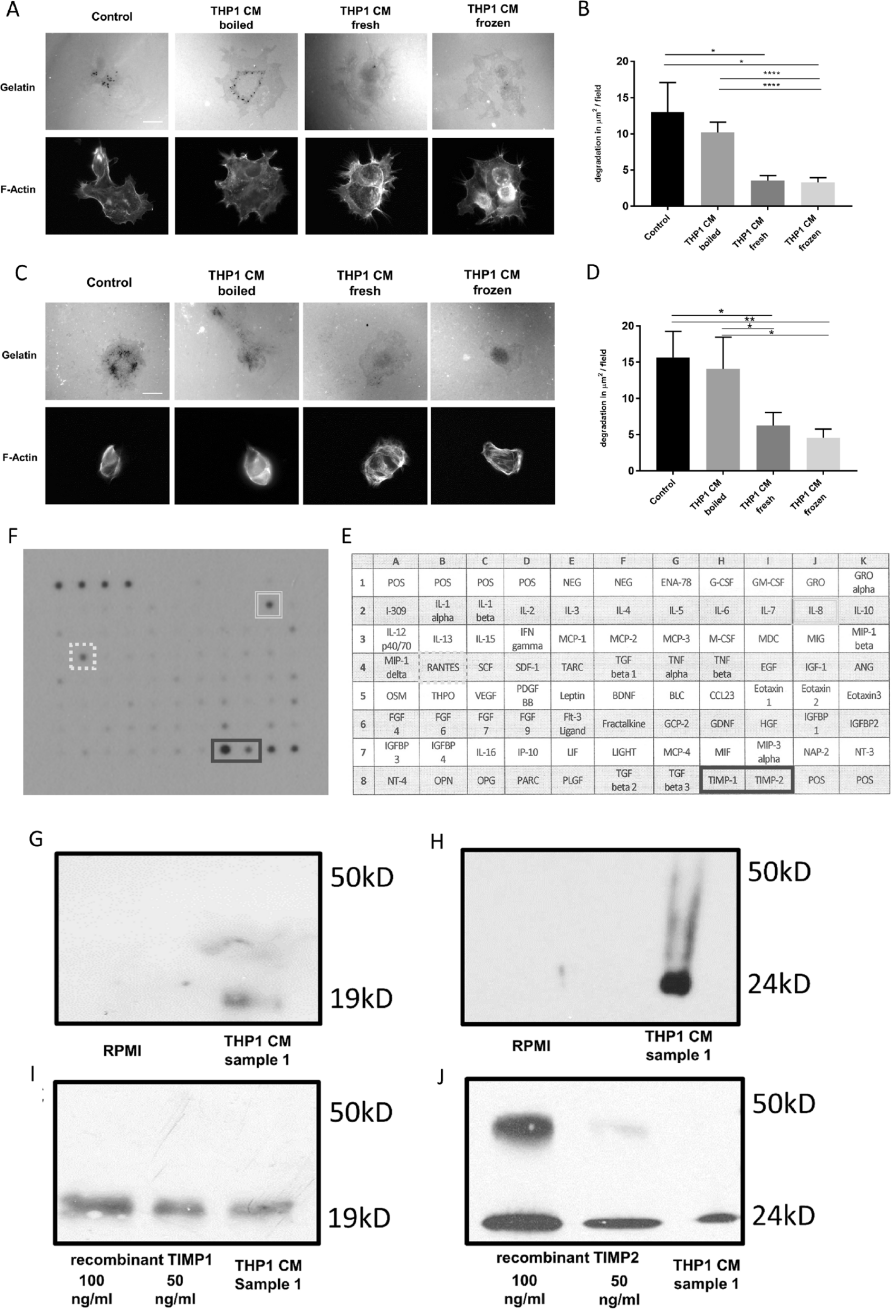
THP1 cells secrete TIMP1 and TIMP2. **a** Representative images from a PaTu8902 invadopodia assay where the cells were either incubated with control medium, conditioned medium that had been applied fresh, boiled or freeze/thawed as indicated. **b** Quantification of degradation per field of view for experimental conditions described above (**a**). **c** Representative images from a CFPAC-1 invadopodia assay where the cells were either incubated with control medium, conditioned medium that had been applied fresh, boiled or freeze/thawed as indicated. **d** Quantification of degradation per field of view for experimental conditions described above (**c**). **e** THP1 conditioned media protein micro array (see methods for details). **f** concentrated samples of THP1 CM and RPMI 1640 control were immunoblotted for TIMP1 using specific antibodies. **g** control media containing recombinant TIMP1 was immunoblotted in comparison to aliquots of concentrated samples of THP1 CM for TIMP1 using specific antibodies. Molecular weight markers indicated for TIMP 1 (19 kD) and TIMP 2 (24 kD). **h** concentrated samples of THP1 CM and RPMI 1640 control were immunoblotted for TIMP2 using specific antibodies. Molecular weight markers indicated for TIMP 1 (19 kD) and TIMP 2 (24 kD). **i** control media containing recombinant TIMP1 was immunoblotted in comparison to a concentrated sample of THP1 CM using TIMP2 using specific antibodies. Molecular weight markers indicated for TIMP 1 (19 kD) and TIMP 2 (24 kD). Experiments were repeated three times. **j** control media containing recombinant TIMP2 was immunoblotted in comparison to a concentrated sample of THP1 CM using TIMP2 using specific antibodies. Note, double bands in Fig. 3j are considered dimers of the TIMP2 molecule. All experiments were repeated three times. Molecular weight markers indicated for TIMP 1 (19 kD) and TIMP 2 (24 kD)

Given that exposure to conditioned medium was sufficient to suppress invadopodia activity we explored this phenomenon in more detail in both cell lines. The gelatin degradation assays were repeated using a control condition (control media) and three different CM conditions. To assess the nature of the inhibitory factor, CM was either boiled, used immediately after collection (fresh CM) or freeze/thawed. Incubation of cells with fresh or freeze/thawed CM significantly reduced invadopodia formation whereas incubation with boiled CM was unable to significantly reduce activity compared to control cells (Fig. 3a-d). These findings suggest that the factors secreted by THP1 cells to suppress invadopodia actively are likely to be protein based.

To further elucidate the nature of THP1 CM suppression of invadopodia activity the THP1 CM was screened against a microarray of selected hormones and cytokines (Fig. 3e). The only proteins detected in significant concentrations were RANTES, Interleukin 8 (IL-8) as well as tissue inhibitor of metalloproteinases 1 and 2 (TIMP1 and TIMP2). Both RANTES [26] and IL-8 are suggested to rather promote than reduce cancer cell invasion, so we focused on the further examination of TIMP1 and TIMP2. Initially, we validated the array by testing whether TIMP1 (Fig. 3g) and TIMP2 (Fig. 3h) were present in the THP1 CM compared to RPMI alone. We then proceeded to determine the concentration of both TIMP1 and TIMP2 in THP CM by comparing the detected signal between the conditioned medium and medium containing known concentrations of recombinant TIMP1 (Fig. 3i) or TIMP2 (Fig. 3j). We were able to establish (see methods section) that there was between 5 and 50 ng/ml of TIMP1 (Fig. 3i) and TIMP2 (Fig. 3j) in THP1 CM.

### Inhibition of invadopodia formation is driven specifically by TIMP2

**Fig. 4:**
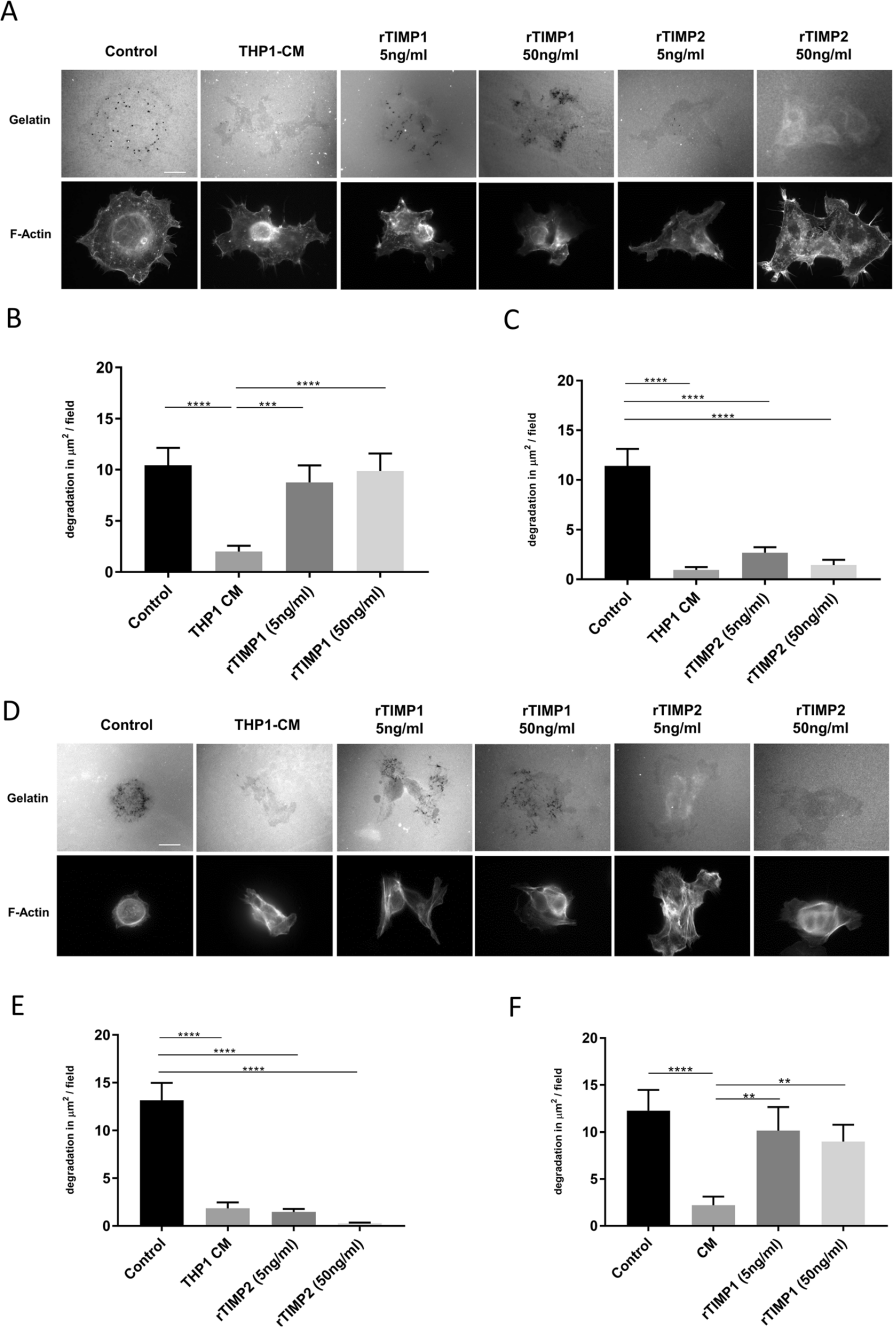
TIMP2 inhibits invadopodia formation in PDAC cells. **a** Representative images from a PaTu8902 invadopodia assay where the cells were either incubated with THP1 conditioned medium (THP1-CM), or were cultured with recombinant TIMP1 (rTIMP1) or recombinant TIMP2 (rTIMP2) at indicated concentrations. **b** and **c** Quantification of degradation per field of view for experimental conditions described above (**a**). **d** Representative images from a CFPAC-1 invadopodia assay where the cells were either incubated with THP1 conditioned medium (THP1-CM), or were cultured with recombinant TIMP1 (rTIMP1) or recombinant TIMP2 (rTIMP2) at indicated concentrations. **e** and **f**) Quantification of degradation per field of view for experimental conditions described above (**d**). In all cases **** = *p* < 0.0001, ** = *p* < 0.01, *** = *p* < 0.001. Experiments were repeated three times

**Fig. 5:**
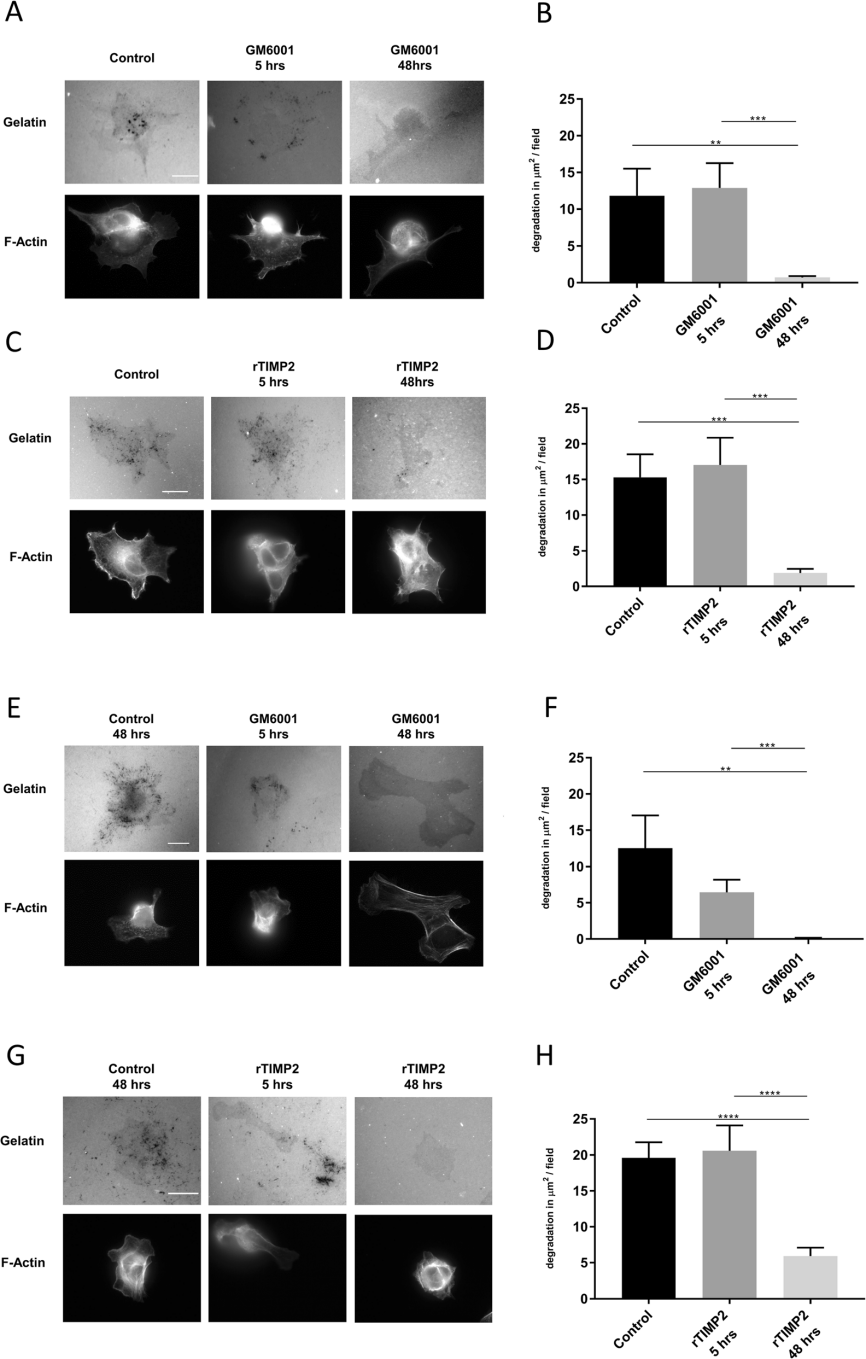
TIMP2-mediated inhibition of invadopodia is reversible after washout in pancreatic cancer cells. **a** Representative images from a PaTu8902 invadopodia assay where the cells were either incubated for 48 h with control medium, control medium with GM6001 (10 μM) washed out after 5 h and replaced with fresh control media or GM6001 (10 μM) left for 48 h. **b** Quantification of degradation per field of view for experimental conditions described above (**a**). **c** Representative images from a PaTu8902 invadopodia assay where the cells were either incubated for 48 h with control medium, control medium with recombinant TIMP2 (rTIMP2, 50 ng/ml) washed out after 5 h and replaced with fresh control medium or control medium with rTIMP2 (50 ng/ml) left for 48 h. **d** Quantification of degradation per field of view for experimental conditions described above (**c**). There was no significant difference in the amount of gelatin degradation between the control cells and the cells where rTIMP2 had been removed post 5 h incubation indicating that PaTu8902 cells were able to recover invadopodia activity once rTIMP2 had been removed. In contrast, again, those cells continuously exposed to rTIMP2 revealed significantly reduced invadopodia formation. **e** Representative images from a CFPAC-1 invadopodia assay where the cells were either incubated for 48 h with control medium, control medium with GM6001 (10 μM) washed out after 5 h and replaced with fresh control media or GM6001 (10 μM) left for 48 h. **f** Quantification of degradation per field of view for experimental conditions described above (**e**). **g** Representative images from a CFPAC-1 invadopodia assay where the cells were either incubated for 48 h with control medium, control medium with recombinant TIMP2 (rTIMP2, 50 ng/ml) washed out after 5 h and replaced with fresh control media or rTIMP2 (50 ng/ml) left for 48 h. **h** Quantification of degradation per field of view for experimental conditions described above. In all cases **** = *p* < 0.0001, *** = *p* < 0.001, ** = *p* < 0.01. Experiments were repeated three times

To assess the specific effects of TIMPs on invasion, the invadopodia assays were repeated adding commercial recombinant TIMP1 (rTIMP1) or recombinant TIMP2 (rTIMP2), respectively at two different concentrations, 5 ng/ml and 50 ng/ml, to cover the range of likely concentration in conditioned medium (Fig. 3i&j). Whilst the presence of rTIMP1 had no impact on degradative ability (Fig. 3a&b), the presence of rTIMP2 was able to significantly reduce gelatin degradation in the treated cells even at a concentration of 5 ng/ml (Fig. 4a&c). Moreover, similar results were obtained when CFPAC-1 cells were treated with rTIMP2 but not with rTIMP1 (Fig. 4d-f). Our results suggest a specific inhibition of invadopodia activity in PDAC cells exposed to low concentrations of TIMP2. However, we cannot rule out a global deleterious effect of rTIMP2 exposure on cell behaviour. To address this issue we tested whether cells could recover invadopodia activity if the rTIMP2 was removed. As a control, responses were compared to incubation with a pharmacological MMP inhibitor GM6001 which is known to transiently inhibit invadopodia formation until washed out [27] (Fig. 5a&b). Subsequently, we tested the recovery of cells incubated with rTIMP2 for 5 h prior to removal (Fig. 5c&d). Similar results were obtained when CFPAC-1 cells were examined in the same protocol (Fig. 5e-h). Thus, the inhibition of invadopodia activity in the presence of rTIMP2 is a specific response. Furthermore, an MTT assay confirmed that neither THP-1 CM nor rTIMP2 had an effect on viability and proliferation (Table 2).

**Table 2:** MTT assay results of PaTu8902 cells with different treatment conditions

	mean % gain in growth	standard deviation
G	52	12
G + SF	52	14
G + CM	52	12
G + rTIMP2 (50 ng/ml)	56	16

G = DMEM supplemented with 10% v/v fetal bovine serum (FBS) and 1 mM penicillin/streptomycin. SF = serum-free DMEM, CM = THP1 conditioned medium, rTIMP2 = recombinant tissue inhibitor of metalloproteinases. ANOVA of raw data reported no significant difference between any of the groups

>

## Discussion

There is evidence suggesting there is a strong link in some tissue types between the ability of cancer cells to form invadopodia in vitro and their invasive potential in vivo [28]. However, only one previous study screened PDAC cell lines for invadopodia formation [25]. Although in this case, the authors reported invadopodia prevalence in most cell lines, this was not comparatively quantified and degradation activity was not assessed. Indeed, we found that some of the PDAC cell lines were extremely inconsistent in invadopodia formation and could not be relied upon for reproducible studies. Other reports focus on one PDAC cell line examining specific pathways [29, 30]. We have now identified two PDAC cell lines that reproducibly potentiate significant invadopodia activity and can be confidently used to further studies in this area. With regards to invadopodia formation, the PDAC cell lines in this study were compared to MDA-MB-231 cells – a breast cancer cell line that is well known for its ability to form invadopodia. Consistent with the findings for different PDAC lines, some breast cancer cell lines are able to form invadopodia (such as MDA-MB-231 or BT-549) [23, 31, 32] whereas others are not (e.g. MCF-7) [33].

Using these cell lines we proceeded to investigate how the TME might impact on invasive activity. The TME has a particular role in PDAC – not only because the major part of PDACs consists of fibroblast and monocytes/macrophages [16] but also because there is a complex interaction between monocytes/macrophages and PDAC cells potentially modifying the invasive potential of PDAC cells [17]. Monocytes and macrophages in the TME appear to have both pro- and antitumor effects which are suggested to be due to different cytokine profiles depending on the differentiation and polarization [34, 35]. Most studies examining mononuclear cells as part of the TME are focusing on macrophages [36, 37], however, there is evidence that monocytes are present in the TME of PDAC [16]. Nevertheless, the prognostic value of monocyte infiltration of the PDAC TME has not been extensively explored.

Taken together, the results of current studies suggest that pro-inflammatory features of the TME determined by the polarization and pro-inflammatory cytokine profile leads to a more aggressive and invasive tumour behaviour and thus decreases prognosis.

This is the first study to show that monocyte-like cells – which are an essential part of the TME [16] – have the ability to reduce the formation of invadopodia in PDAC cells and thus highly significantly decrease their invasive migratory behavior, which would inhibit their metastatic potential. Interestingly, THP-1 cells are able to degrade the ECM and form invadopodia as well [38].

Our results indicate that the suppressive activity of undifferentiated monocyte-like cells of the PDAC cells might be mediated via secretion of TIMP2 but not TIMP1. This is consistent with a report in breast cancer cells that suggested TIMP2 suppressed invadopodia formation. However, in this study, activity was measured by positive cortactin staining but not matrix degradation [13]. The specific inhibitory effect of TIMP2 might be due to the differential inhibition of TIMP1 and TIMP2. Indeed, MMP2, MMP9, and MT1-MMP are considered the most important MMPs needed for invadopodia activity [39]. MT1-MMP, a membrane-bound MMP, has a key role not only degrading the ECM but also activating of MMP2 [40] which in turn activates MMP9 [41]. In contrast, TIMP1 inhibits soluble MMPs and has a very low affinity to membrane-bound MMPs such as MT1-MMP. Taken together, there is evidence that the TIMP2-mediated inhibition of MT1-MMP is the key to inhibiting invadopodia formation [42]. Nonetheless, we believe that there are other mechanisms of how undifferentiated monocyte-like cells can inhibit invadopodia formation as well since we found that a monocyte-like cell/PDAC cell co-culture prior to the actual invadopodia assay was also able to reduce cancer cell invasion (Fig. 1c and d). These mechanisms could include transcriptional changes in the cancer cells, e.g. upregulation of S100A8 and S100A9 which is known to increase the invasive potential of cancer cells [43].

Interestingly, in human hepatocellular carcinoma (HCC) samples, downregulation of TIMP2 expression was significantly associated with liver invasion and poorer survival outcomes [44]. Currently, PDAC tumours are considered to be immunologically “cold” [45]. We would suggest that an aspiration to increase the immune infiltrate in the PDAC setting [46] should consider specifically increasing the presence of TIMP2 secreting monocyte-like cells.

We found that THP1 monocyte-like cells produce TIMP2 in relevant concentrations without being co-cultured with PDAC cells. THP1 CM is able to suppress invadopodia formation, a direct interaction between THP1 cells and PDAC cells is not necessary for this effect. However, there is evidence that a direct co-culture of mononuclear cells and cancer cells leads to changes both in MMP and TIMP expression levels [47]. Besides the examination of these interactions, further experiments could also use human monocyte derived macrophages in the stages of M0, M1, and M2 to see the differential effects on invasion depending on the polarisation of the macrophages. To translate these findings into an in vivo model, monocyte-like cells or differentiated macrophages could be injected and isolated in nude/SCID mice.

## Conclusions

In conclusion, our findings are indicative that TIMP2 could both be a potential prognostic marker and a therapeutic target in PDAC. A high ratio of TIMP2-secreting monocyte-like cells could be associated with reduced metastatic potential and better prognosis. Furthermore, the application of synthetic TIMP2 agonists could possibly lead to a reduction of cancer cell invasion in vivo.

## Data Availability

The datasets generated during and/or analysed during the current study are available from the corresponding author on reasonable request.

## Abbreviations

CM: Conditioned media
CO_2_: Carbon dioxide
DAPI: 4′,6-Diamidino-2-Phenylindole
DC: Direct co-culture
DMEM: Dulbecco’s Modified Eagle Medium
DMFZ: Deutsche Sammlung von Mikroorganismen und Zellkulturen GmbH
ECM: Extracellular matrix
eGFP: Enhanced green fluorescent protein
FBS: Fetal bovine serum
GAPDH: Glyceraldehyde 3-phosphate dehydrogenase
GM: Growth medium
HCC: Hepatocellular carcinoma
HRP: Horseradish peroxidase
MMP 2/9/14: Matrix metalloproteinase 2/9/14
MT-MMP1: Membran-type matrix metalloproteinase 1
MTT: 3-(4,5-dimethylthiazol-2-yl)-2,5-diphenyltetrazolium bromide
PBS: Phosphate Buffered Saline
PC: Previous co-culture
PDAC: Pancreatic ductal adenocarcinoma
RPMI: Roswell Park Memorial Institute 1640 Medium
RT: Room temperature
rTIMP1/2: Recombinant tissue inhibitor of matrix metalloproteinases 1/2
SDS: Sodium dodecyl sulfate
TAM: Tumour-associated macrophages
TIMP1/2: Tissue inhibitor of matrix metalloproteinases 1/2
TME: Tumour microenvironment
